# Conventional liquid-based techniques versus Cytyc Thinprep^® ^processing of urinary samples: a qualitative approach

**DOI:** 10.1186/1472-6890-5-9

**Published:** 2005-10-06

**Authors:** Eric Piaton, Jacqueline Faÿnel, Karine Hutin, Marie-Claude Ranchin, Michèle Cottier

**Affiliations:** 1 INSERM U.407/Université Claude Bernard Lyon 1, Faculté de Médecine Lyon Sud, 69495 Pierre Bénite Cedex, France; 2 Laboratoire de Cytopathologie, Hôpital Edouard Herriot, Place d'Arsonval, 69437 Lyon Cedex 03, France; 3 Laboratoire d'Histologie, CHRU de Saint-Etienne, Hôpital Nord, 42055 Saint-Etienne Cedex 2, France

## Abstract

**Background:**

The aim of our study was to objectively compare Cytyc Thinprep^® ^and other methods of obtaining thin layer cytologic preparations (cytocentrifugation, direct smearing and Millipore^® ^filtration) in urine cytopathology.

**Methods:**

Thinprep slides were compared to direct smears in 79 cases. Cytocentrifugation carried out with the Thermo Shandon Cytospin^® ^4 was compared to Thinprep in 106 cases, and comparison with Millipore filtration followed by blotting was obtained in 22 cases. Quality was assessed by scoring cellularity, fixation, red blood cells, leukocytes and nuclear abnormalities.

**Results:**

The data show that 1) smearing allows good overall results to be obtained, 2) Cytocentrifugation with reusable TPX^® ^chambers should be avoided, 3) Cytocentrifugation using disposable chambers (Cytofunnels^® ^or Megafunnel^® ^chambers) gives excellent results equalling or surpassing Thinprep and 4) Millipore filtration should be avoided, owing to its poor global quality. Despite differences in quality, the techniques studied have no impact on the diagnostic accuracy as evaluated by the rate of abnormalities.

**Conclusion:**

We conclude that conventional methods such as cytocentrifugation remain the most appropriate ones for current treatment of urinary samples. Cytyc Thinprep processing, owing to its cost, could be used essentially for cytology-based molecular studies.

## Background

More than 50,000 new cases of urothelial carcinoma, which represents 90% of bladder cancer cases are diagnosed annually in Europe and in North America [1]. About 70% of bladder urothelial carcinomas are superficial (TNM stage pTa-1) and may be viewed, diagnosed and treated by cystoscopy aided by biopsies and transurethral resection [2].

Despite it is recognized as the biological standard for the diagnosis and follow up of bladder tumors, urinary cytology has a mean sensitivity of about 50% and it is hampered by a large amount of non-diagnostic samples [3]. Although urinary cytology detects about 80% of aggressive, high grade (G3) urothelial tumors, some results remain falsely negative, particularly in patients having had TUR or bacillus Calmette-Guérin immunotherapy. In urology practice, cystoscopy is commonly combined with urinary cytology, particularly in the search for high grade wherever its location in the urinary tract.

Liquid-based cytology (LBC) has been developed as a replacement to cytocentrifugation and/or smearing, owing to cell recovery capabilities and better cell preservation. Some LBC methods use a filtration process and a computer-assisted thin-layer deposition of cells (Cytyc Thinprep^® ^supplied by Cytyc Corp., Boxborough, MA), whereas others are based on a sedimentation process (AutoCyte^® ^PREP supplied by TRiPath Imaging, Burlington, NC). In the urine, the use of Cytyc Thinprep 2000 results in increased cellularity and marked reduction of debris, red blood cells (RBC) and crystals [4-7].

However, optimization of cell capture and fixation as well as thin-layer deposition of cells can be achieved by other methods than LBC, particularly while using modern cytocentrifugation methods [7]. In our experience based on 2500 specimens/year for 15 years, and provided specific requirements are followed, direct smears and cytocentrifugation with the Shandon Cytospin^® ^4 (Thermo Electron Corp., Waltham, MA) produce highly satisfying cytological specimens.

Accordingly, the aim of our study was 1) to objectively analyze the quality of urine samples processed by a body of conventional thin-layer methods as compared with Cytyc Thinprep LBC and 2) to verify if differences noted have an impact on diagnostic accuracy.

## Methods

The study population was composed of 224 urine samples taken in patients with symptoms suggesting bladder cancer (gross hematuria, micturition disorders, chronic urinary infection) in 89 cases (39.7%), or followed after transurethral resection for bladder urothelial carcinoma in 135 cases (60.3%).

Urinary samples were taken after cystoscopy in 157 cases (63.8%), and after simple micturition in other cases. All samples were immediately fixed with 50% ethanol (V/V) or with a 20% Polyethyleneglycol 1500 (Merck, Darmstadt, Germany) solution in 50% ethanol (1/3 fixative and 2/3 urine).

Urine samples were sent to the laboratory and separated into two aliquots after homogeneization. One of the aliquots was processed according to the Thinprep LBC recommendations, and the other was processed according to a smear method, by cytocentrifugation or by filtration.

### Cytyc Thinprep* processing

The Thinprep 2000 automaton allows thin-layer cell preparations to be provided thanks to a filtration process: after the TransCyt^® ^filter has been plunged into the sample, it rotates at a high speed and facilitates cell and mucus dispersion. A vacuum is then applied to the filter, which collects cells on a 5 μm porosity membrane. A software program allows a homogeneous deposition of cells until saturation. The TransCyt filter is then inverted and a positive pressure allows cells to adhere to an electronegative slide. After insertion of another TransCyt filter and of another slide, the whole procedure may be repeated until the entire sample has been treated.

The urine samples studied were processed according to instructions for non mucoid fluids: samples were mixed with a Cytolyt^® ^solution containing methanol, mucolytic and hemolytic agents and were then centrifuged at 600 G for 10 minutes. After discarding the supernatant, the cell pellet was mixed with a PreservCyt^® ^solution and treated by the Thinprep 2000 processor. Thinprep slides were used in all cases.

### Smearing on coated slides

Comparison of LBC with smears was made in 79 cases. After centrifugation at 600 G for 10 minutes and careful removing of the supernatant, the cell pellet was aspirated and smeared on a thin coating layer (Glycerin/Albumin according to Mallory, Bayer Diagnostics, Puteaux, France) previously deposited on two Superfrost^® ^Plus slides (Menzel-Gläser, Braunschweig, Germany). Slides were immediately fixed with a Cell-Fixx^® ^(Thermo Electron Corp., Waltham, MA) spray and allowed to dessicate at room temperature (RT) for at least 1 hour before Papanicolaou staining.

### Cytocentrifugation methods

Comparison of LBC with cytocentrifugation was made using the Thermo Shandon Cytospin^® ^4 in 106 cases. After centrifugation at 600 G for 10 minutes, hypocellular urine samples (< 20 μl cell pellets) were cytocentrifuged with sample chambers up to 0.5 ml. Conversely, urine samples with a large pellet were treated with large volume sample chambers.

The Cytospin system uses centrifugation and fluid absorption principles and allows deposition of a thin layer of cells on round or rectangular areas. The deposition process needs that sample chambers are placed and locked into stainless steel Cytoclip^® ^assembly devices. In order to test various types and qualities of sample chambers we used:

1) three years' old round reusable, autoclavable chambers designed for samples up to 0.5 ml (TPX^® ^chambers with a cell deposition area of 6 mm diameter, allowing 28 mm^2 ^to be screened) in 44 cases,

2) round disposable chambers designed for samples up to 0.5 ml (single Cytofunnel^® ^with a cell deposition area of 6 mm diameter, allowing 28 mm^2 ^to be screened) in 31 cases,

3) large volume disposable chambers designed for samples up to 6 ml (Megafunnel^® ^chambers with a cell deposition area of 21 × 24 mm, allowing 294 mm^2 ^to be screened) in 31 cases.

Two slides of 28 mm^2 ^screening area (for 1 ml of urine), and one slide of 294 mm^2 ^screening area (for 6 ml of urine) were prepared for each specimen studied.

Specially marked coated Cytoslides^® ^provided by Thermo Shandon were used. Although not necessary, slides processed with TPX sample chambers had an additional treatment with a drop of glycerine/albumin deposited on the sample area.

### Millipore filtration methods

LBC was compared with Millipore filtration followed by blotting of cells on various slides in 39 cases, in order to test the adhesiveness to various types of commercially available coated slides. Urine was filtered through Magna^® ^MCE nitrocellulose membrane filters, pore size 5 μm, diameter 25 mm placed in a Swinnex^® ^device attached to a 60 ml Luer-Lock^® ^syringe (Bioblock Scientific, Illkirch, France).

After complete filtration and removal of the membrane filter, the blotting was first performed on Polysine^® ^slides (Menzel-Gläser, Braunschweig, Germany) in 8 cases, but the adhesiveness obtained was too impaired for allowing continuation of the assays. We then used Cytyc Thinprep slides in 9 cases, but finally we chose Superfrost^® ^Plus slides and Snowcoat X-tra^® ^slides (Surgipath Europe Ltd, Peterborough, England) equally for the 22 remaining cases.

Using these procedures, the resulting cell deposition area is 25 mm diameter, allowing about 491 mm^2 ^to be screened.

Smears were stained with a hypochromic Papanicolaou stain [8] before analysis.

### Analysis of morphologic criteria

A single pathologist (EP) compared conventional and LBC slides using an Olympus BHS microscope. Slides were placed side by side and were analyzed under Plan × 10, Plan × 40 and Oil PlanApo x63 objectives. The global quality of slides was assessed by scoring cellularity, cell fixation, number of RBC, leukocytes and degenerative changes of urothelial cells. The presence of cell groups and clusters was also measured. Special attention was paid to altered cellular features potentially indicating malignant transformation – increased N/C ratio, nuclear hyperchromatism, irregular nuclear shape, prominent nucleoli and mitoses – as previously described [9].

All cellular features were coded from 0 to xxx according to their degree of abnormality.

Urothelial cells were recognized as malignant, high-grade, when they showed increased N/C ratio, nuclear hyperchromatism and markedly irregular nuclear borders or prominent nucleoli. They were recognized as neoplastic, low-grade, when they formed papillary fronds demonstrating increased N/C ratio and slightly irregular nuclear shape, or where numerous elongated cells with slight nuclear abnormalities could be evidenced, as described in the literature [9,10].

Cytological results were categorized as positive or negative for urothelial tumor cells, whatever their grade. Normal, inflammatory, reactive and degenerative conditions of urothelial cells were considered as negative, as well as urothelial atypias of undetermined significance.

Numerical data were analyzed using paired series Chi-square test or Fisher's exact test, when appropriate, and a probability level of 0.05 was regarded as significant.

## Results

**Figure 1:**
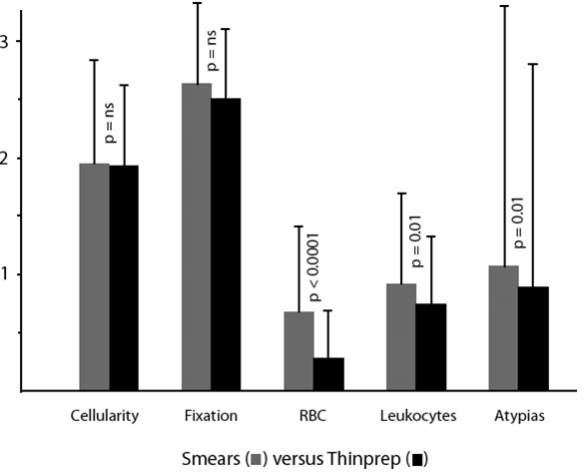
Comparison of smears versus Thinprep slides (mean values, standard deviations and statistical significance).

**Figure 2:**
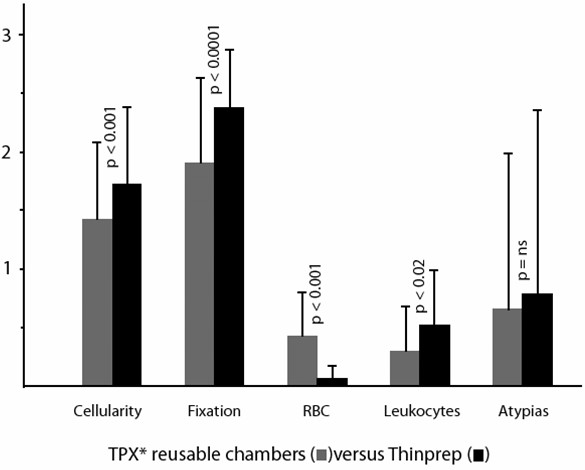
Comparison of cytocentrifugation using reusable TPX chambers versus Thinprep slides (mean values, standard deviations and statistical significance).

**Figure 3:**
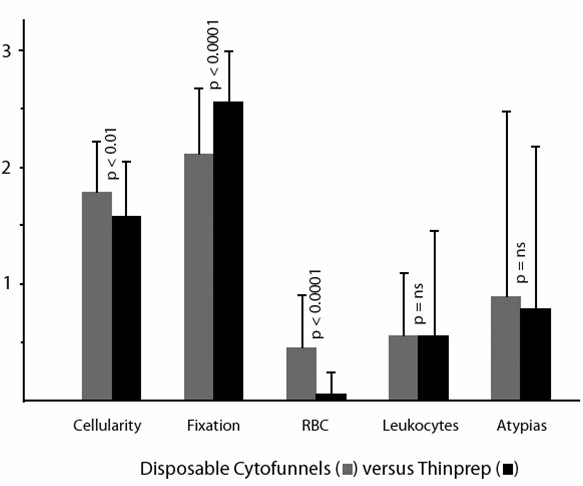
Comparison of cytocentrifugation using disposable Cytofunnels (for samples up to 0.5 ml) versus Thinprep slides (mean values, standard deviations and statistical significance).

**Figure 4:**
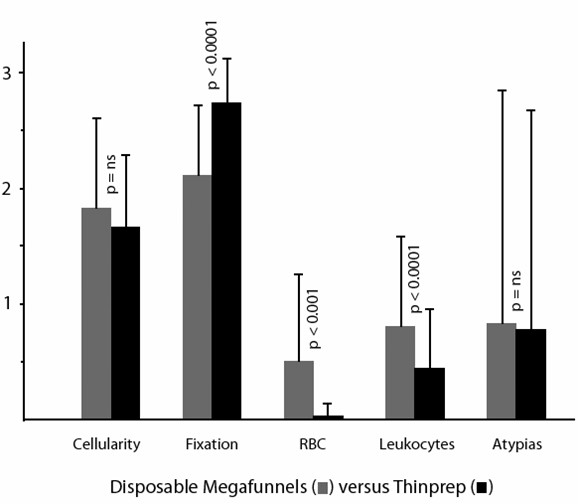
Comparison of cytocentrifugation using disposable Megafunnels (for samples up to 6 ml) versus Thinprep slides (mean values, standard deviations and statistical significance).

**Figure 5:**
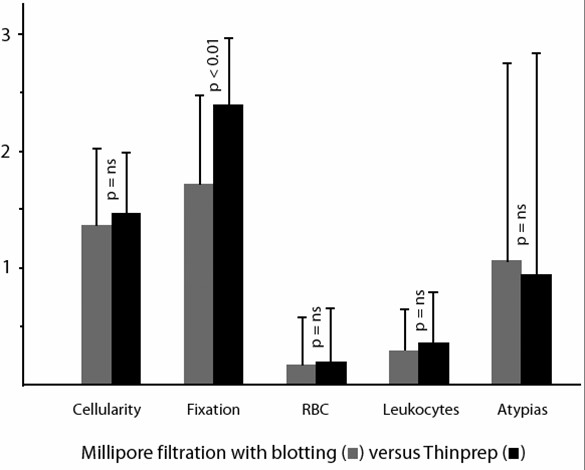
Comparison of Millipore filtration followed by blotting of cells on coated slides versus Thinprep slides (mean values, standard deviations and statistical significance).

Using the scoring system as described in the Materials and Methods section, and considering a 0–3 scale, mean and standard deviations as well as the statistical significance of each parameter are shown in Figure 1,2, 3, 4, 5.

**Figure 6:**
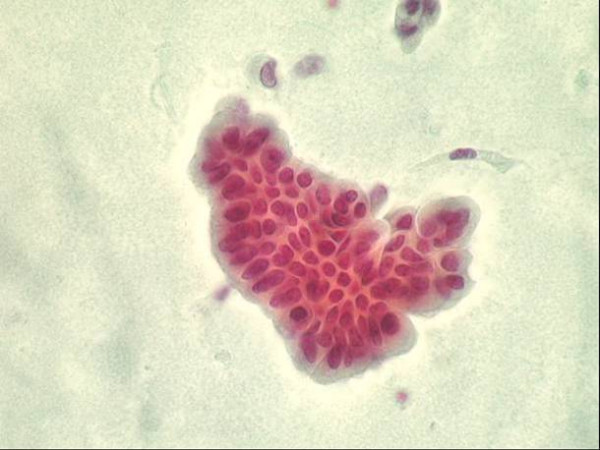
Group of low grade Urothelial tumour cells obtained after centrifugation and smearing. Papanicolaou stain, x 400.

Differences noted concern global quality (cellularity and fixation combined) on the one hand, number of RBC and leukocytes on the other hand. Surprisingly, we found that smears allowed obtaining a global quality superimposable to that of Cytyc Thinprep slides (Figure 6). More precisely, the cellularity scores obtained by smears and LBC were 1.97 ± 0.86 versus 1.96 ± 0.68, respectively (p = ns), whereas values for fixation were 2.58 ± 0.48 versus 2.50 ± 0.59 (p = ns).

**Figure 7:**
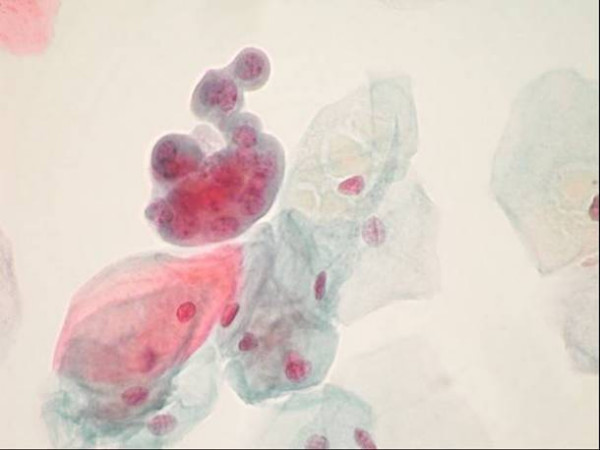
Group of low grade urothelial tumour cells together with normal superficial cells obtained after liquid based (Thinprep) processing. Papanicolaou stain, x 630.

Cytocentrifugation with 3 years' old reusable sample chambers resulted in significant decrease in both cellularity and fixation quality, whereas cytocentrifugation with disposable sample chambers (whatever the type of chamber used) allowed obtaining the better results (Figure 7).

Millipore filtration resulted in impaired cell preservation even after blotting of cells on coated slides and careful fixation.

The concentration of RBC was significantly decreased after LBC treatment of samples in all circumstances except in Millipore* filtration using 5 μm porosity membranes. Similar comments may be done about leukocytes.

Whatever the technique studied, the search for cell groups and atypias gave results identical than those of LBC except for smears which showed a slightly higher percentage (p = 0.01). However, the values obtained were not strikingly different.

## Discussion

As far back as the late 'seventies, authors have attempted to compare cytocentrifugation with other methods such as filtration [11,12]. In those preliminary studies, Millipore filtration was found to give better cell recovery and better morphologic details than cytocentrifugation. However the methods used (reusable sample chambers) was suboptimal: a significant cell loss can be attributed to the roughness of sample chamber walls secondary to repeated cleaning [13].

Waiting the 'nineties was necessary for obtaining comparisons between the Cytyc Thinprep LBC and other methods, with some contradictory results. Many of the studies, published as abstracts of the 40^th ^and 41^st ^Annual Scientific Meetings of the International Academy of Cytology, were not transformed into full length articles [4,5,14,15].

Except for one study which showed processing time and cost several times greater for Cytyc Thinprep LBC than for polycarbonate membrane filtration [15], most series recognize advantages in using LBC. In a recent study comparing cytocentrifugation to Cytyc Thinprep, Cytospin preparations were found superior to LBC in terms of cytomorphologic details and preservation of architectural patterns [16]. However the advantage of LBC concerning cleaner background was noted.

Cytocentrifugation and LBC are not the only available methods for improving diagnostic accuracy: potentially interesting results were previously shown by Albright and Frost [17]. Using a simple density gradient to separate atypical cells from normal cells after fixation with the Saccomanno method, the authors were able to enrich up to 20-fold the atypical and cancer cell fraction. To our knowledge however, these results have not been resumed at a later date.

A more recent study assessed the quality and cost of AutoCyte PREP versus cytocentrifugation of urine specimens in a general laboratory setting [18]. It was shown that the Cytospin method, despite longer preparation time, had 1) shorter screening time, 2) higher number of diagnostic cells, 3) better fixation and staining quality than the AutoCyte PREP. Additionally, the Cytospin method was found 7 times less expensive than the AutoCyte* PREP method.

Concerning conventional methods, the values obtained in our series show that despite differences in quality, the techniques studied have no impact on the diagnostic accuracy as evaluated by the rate of abnormalities (nuclear features and cell groups). About each technique studied, the following comments may be done:

1. Smearing allows obtaining good overall results for the lowest cost. However the longer screening time renders the method suboptimal. Additionally the glycerine/albumin coating used renders slides useless for immunocytochemistry or other molecular studies,

2. Cytocentrifugation with reusable chambers should be avoided if annual renewal cannot be guaranteed,

3. Millipore filtration followed by blotting of cells on coated slides should be avoided, owing to poor global quality and high cost,

4. Cytocentrifugation using disposable chambers (Cytofunnels or Megafunnel chambers) gives excellent results equalling or surpassing LBC if one considers cellularity, fixation and the comfort for screening.

Concerning cost-efficacy comparisons, it has been shown that the monthly cost of the two most efficient methods (Cytocentrifugation with disposable chambers and Cytyc Thinprep LBC) is strikingly different: there is a 92.8% to 154.5% increased cost for LBC versus cytocentrifugation with disposable Megafunnels and Cytofunnels, respectively [19].

However in our opinion, one must consider not only the diagnostic performance and cost, but also the ultimate goal of technical improvements provided by LBC. LBC aims primarily to provide reproducible and well preserved material for additional techniques such as immunocytochemistry, fluorescence in situ hybridization (FISH) and other types of molecular analyses. It has been shown that Thinprep-processed samples allowed efficient recovery of the DNA, RNA and proteins related to the p53 tumor suppressor gene [20].

## Conclusion

We conclude that Cytyc Thinprep LBC, despite its cost, may still be considered as a technical progress for cytology-based molecular studies. To an economical point of view and taking into account the value of a meticulous technique, cytocentrifugation with disposable chambers remains the technical standard for current treatment of urinary samples.

## List of abbreviations

LBC: liquid-based cytology technique

RBC: red blood cells

RT: room temperature

TUR: transurethral resection

## Competing interests

The author(s) declare that they have no competing interest.

## Authors' contributions

EP planned the study and prepared the manuscript.

JF, KH and MCR performed the liquid-based techniques (cytocentrifugations and Thinprep processing) as well as smears and Millipore filtrations.

EP and MC performed the cytopathologic evaluations and the statistical analysis All authors read and approved the final manuscript.

## Pre-publication history

The pre-publication history for this paper can be accessed here:


